# Production efficiency of the bacterial non-ribosomal peptide indigoidine relies on the respiratory metabolic state in *S. cerevisiae*

**DOI:** 10.1186/s12934-018-1045-1

**Published:** 2018-12-13

**Authors:** Maren Wehrs, Jan-Philip Prahl, Jadie Moon, Yuchen Li, Deepti Tanjore, Jay D. Keasling, Todd Pray, Aindrila Mukhopadhyay

**Affiliations:** 1 Biological Systems and Engineering Division, Lawrence Berkeley National Laboratory, Berkeley, CA 94720 USA; 2 Institut für Genetik, Technische Universität Braunschweig, Brunswick, Germany; 3 Joint BioEnergy Institute, Lawrence Berkeley National Laboratory, Emeryville, CA 94608 USA; 4 Advanced Biofuels and Bioproducts Process Development Unit, Lawrence Berkeley National Laboratory, Emeryville, CA 94608 USA; 5 Department of Plant and Microbial Biology, University of California, Berkeley, CA 94720 USA; 6 Department of Bioengineering, University of California, Berkeley, CA 94720 USA; 7 Department of Chemical and Biomolecular Engineering, University of California, Berkeley, CA 94720 USA; 8 The Novo Nordisk Foundation Center for Biosustainability, Technical University of Denmark, Kongens Lyngby, Denmark; 9 Synthetic Biochemistry Center, Institute for Synthetic Biology, Shenzhen Institutes for Advanced Technologies, Shenzhen, China; 10 Environmental Genomics and Systems Biology Division, Lawrence Berkeley National Laboratory, Berkeley, CA 94720 USA

**Keywords:** Non-ribosomal peptide synthetase, NRPS, *S. cerevisiae*, Indigoidine, Metabolic state, TCA cycle activity, Non-ribosomal peptide synthesis, Bioreactor, BpsA, BJ5465

## Abstract

**Background:**

Beyond pathway engineering, the metabolic state of the production host is critical in maintaining the efficiency of cellular production. The biotechnologically important yeast *Saccharomyces cerevisiae* adjusts its energy metabolism based on the availability of oxygen and carbon sources. This transition between respiratory and non-respiratory metabolic state is accompanied by substantial modifications of central carbon metabolism, which impact the efficiency of metabolic pathways and the corresponding final product titers. Non-ribosomal peptide synthetases (NRPS) are an important class of biocatalysts that provide access to a wide array of secondary metabolites. Indigoidine, a blue pigment, is a representative NRP that is valuable by itself as a renewably produced pigment.

**Results:**

*Saccharomyces cerevisiae* was engineered to express a bacterial NRPS that converts glutamine to indigoidine. We characterize carbon source use and production dynamics, and demonstrate that indigoidine is solely produced during respiratory cell growth. Production of indigoidine is abolished during non-respiratory growth even under aerobic conditions. By promoting respiratory conditions via controlled feeding, we scaled the production to a 2 L bioreactor scale, reaching a maximum titer of 980 mg/L.

**Conclusions:**

This study represents the first use of the *Streptomyces lavendulae* NRPS (BpsA) in a fungal host and its scale-up. The final product indigoidine is linked to the activity of the TCA cycle and serves as a reporter for the respiratory state of *S*. *cerevisiae*. Our approach can be broadly applied to investigate diversion of flux from central carbon metabolism for NRPS and other heterologous pathway engineering, or to follow a population switch between respiratory and non-respiratory modes.

**Electronic supplementary material:**

The online version of this article (10.1186/s12934-018-1045-1) contains supplementary material, which is available to authorized users.

## Background

Microbial metabolic pathway discovery and engineering efforts have led to an increasing number of biotechnological processes in diverse sectors of our economy, ranging from energy to health and medicine, as well as food and agriculture. Industrial-scale microbial production environments are profoundly different from the cultivation environments commonly used at lab scale. Thus, beyond pathway engineering, understanding microbial physiology in these different environments is essential to translate proof-of-concept bioprocesses from shake flasks to industrially-relevant bioreactor setups [1, 2]. During large-scale biotechnological production processes, insufficient mixing commonly leads to micro-environmental inhomogeneities with severe concentration gradients of important cultivation characteristics, particularly dissolved oxygen and carbon sources [3]. The benefit of using facultative anaerobic microbes in industrial processes arises from their ability to switch between fermentative and respiratory metabolism to produce ATP depending on the availability of oxygen without loss of viability. However, fluctuations in dissolved oxygen and carbon sources are recognized to trigger metabolic and transcriptional responses, with unfavorable effects on productivity [2–4].

**Fig. 1:**
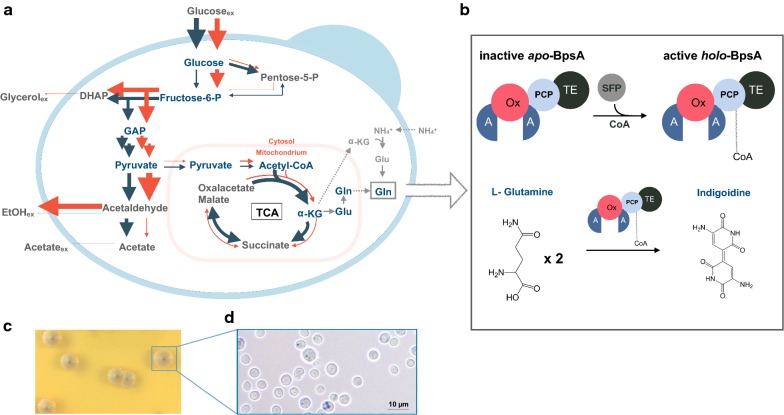
Production of bacterial indigoidine in engineered *S. cerevisiae*. **a** *S. cerevisiae* exhibits two distinct metabolic states which are accompanied with distinct metabolic flux profiles. The width of the arrows represents metabolic flux. Blue arrows represent purely respiratory state, while red arrows represent fully fermentative state. *GAP* glyceraldehyde 3-phosphate, *DHAP* dihydroxyacetone phosphate, *EtOH* ethanol, *α-KG* α-ketoglutarate, *Glu* glutamate, *Gln* glutamine. Several known pathways for glutamine biosynthesis are shown. The depiction of metabolite intermediates and their cellular localization adapted from Frick et al. Ljungdahl and Daignan-Fornier, and Chen et al. [10, 48, 49]. **b** Activation of the apo-form of the *S. lavendulae* NRPS, BpsA (blue pigment synthetase A) by the *Bacillus subtilis* 4′-phosphopantetheinyl transferase (PPTase; Sfp) via addition of a coenzyme A-derived moiety to the peptide carrier domain (PCP) into the active holo-form. The active holo-BpsA converts two l-glutamines to one molecule of the blue pigment indigoidine by a catalytic process involving adenylation (**a**), oxidation (Ox) and thioesterase (TE) domains. **c** Positive *S. cerevisiae* transformants exhibit blue pigmentation occurring 3 days after visible colony formation on solid media containing glucose. **d** Brightfield microscopy of the pigmented colony shows heterogeneity in pigment production, ×63 zoom. The pigment shows punctate subcellular localization, scale bar = 10 µm, increasing non-linear magnification of boxed areas is depicted by pull-outs

*Saccharomyces cerevisiae* is not only used extensively for proof-of-concept pathway studies but also as a host for many applied industrial processes [5, 6]. In contrast to many other fungal or bacterial hosts, *S. cerevisiae* adjusts its energy metabolism based on the nature of available carbon sources via carbon catabolite repression [7]. Even under aerobic conditions, *S. cerevisiae* predominantly metabolizes glucose by fermentation leading to the production of ethanol, glycerol and carbon dioxide (Fig. 1a, red arrows) [8, 9]. Upon glucose depletion, the non- fermentable products of fermentation ethanol and glycerol can serve as carbon sources, requiring a shift to respiratory mode. The metabolic shift from fermentative to respiratory growth is accompanied by changes of carbon flux and gene expression throughout the whole central metabolism [10, 11]. Under purely fermentative conditions, a redirection of metabolic flux from the tricarboxylic acid (TCA) cycle towards fermentative pathways results in a low activity of the TCA cycle. When switching from fermentative to respiratory conditions, the flux to the TCA cycle increases significantly to enable respiration (Fig. 1a, blue arrows) [10, 12, 13]. Thus, activity of the TCA cycle presents an appropriate proxy to distinguish metabolic states in *S. cerevisiae* [14]. While the effect of the metabolic state on native pathways and products has been investigated [15–18], its effect on engineered pathways and biosynthetic products remains understudied.

In this study, we engineered *S. cerevisiae* for the production of indigoidine, a non-ribosomal peptide synthetase (NRPS) derived compound, formed by condensation of two l-glutamine residues. Specifically, we use the bacterial Blue pigment synthetase (BpsA) from *Streptomyces lavendulae* [19], that has not been expressed in a fungal host before. Non-ribosomal peptides present a diverse class of secondary metabolites with various important biological activities. Indigoidine itself, provides a renewably produced pigment for the dye industry that has reinvigorated its search for environmentally friendly processes [20]. Indigoidine is an ideal final heterologous product to examine the importance of respiratory and non-respiratory environments as its precursor pool is linked to the TCA cycle. We examine the effect of the metabolic state on this heterologous product derived from the TCA cycle, a pathway highly responsive to metabolic shifts. Using colorimetric production assays and metabolomics, we demonstrated that the production of the indigoidine is connected to the metabolic state of the cell and can be maintained with high fidelity if *S. cerevisiae* is kept in respiratory mode. Further, we use this knowledge to maintain high levels of indigoidine production when transitioning between cultivation format and scales.

## Results and discussion

### Establishing indigoidine production in *Saccharomyces cerevisiae*

In *S. lavendulae*, the native pathway to convert l-glutamine into the blue pigment indigoidine consists of the NRPS BpsA and a 4′-phosphopantetheinyl transferase (PPTase), needed to activate the apo-NRPS into its holo-form via the addition of a coenzyme A-derived phosphopantetheine moiety (Fig. 1b) [19, 21]. To establish the indigoidine pathway in *S. cerevisiae,* we genomically integrated the *Bacillus subtilis* PPtase *sfp*, previously shown to successfully activate apo-BpsA [22], and the 3.8 kbp NRPS gene *bpsA* into *S. cerevisiae* BJ5465, a protease deficient strain reported to functionally express Sfp [23].

Blue pigment production was successfully observed in the resulting strain 3 days after visible colony formation (Fig. 1c). The pigmentation appeared first in the central colony region and was limited to subpopulations on the colony surface and extended outwards of the colony over the course of 10 days (Additional file 1: Figure S1). This observation indicates that the localization of a given cell within a colony has an effect on the production. This effect could originate from enhanced oxygen availability at the surface of the central colony region as compared to the outer limits or lower layers of a colony [24], as oxygenation is a necessary step in the formation of the pigment [25].

To determine the localization of the pigment within the cell, we performed brightfield microscopy of the transformants. As expected from the phenotype of the colony, the population shows heterogeneity regarding pigment production (Fig. 1d). In cells that produce the blue pigment, it accumulates in foci and forms aggregates.

### Carbon source determines efficiency of indigoidine production

While glucose is its preferred carbon source, *S. cerevisiae* can utilize other sugars such as sucrose, galactose and a variety of non-fermentable substrates including glycerol by adjusting its energy metabolism from fermentation to respiration. The flux through the TCA cycle is significantly increased during respiration compared to that during fermentation (Fig. 1a) [10]. The TCA cycle intermediate alpha-ketoglutarate serves as an indirect precursor pool for indigoidine formation via the amino acids glutamate and glutamine.

**Fig. 2:**
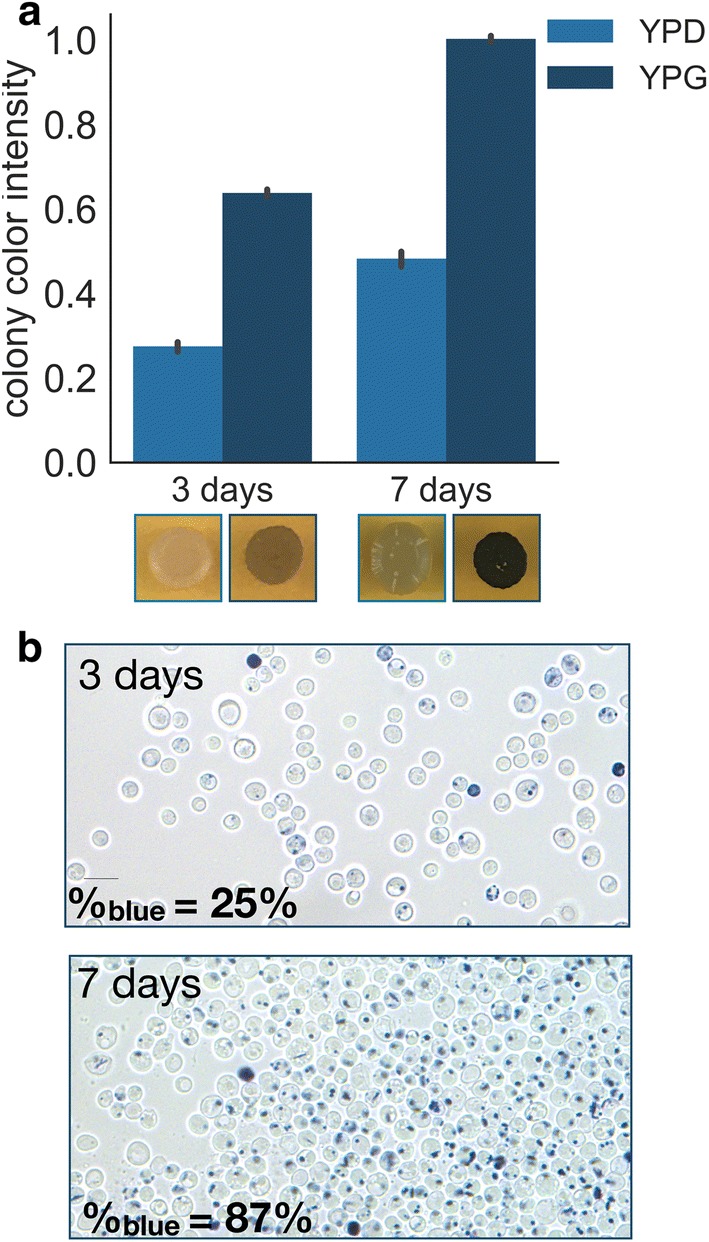
Phenotype of BJ5465.sfp.bpsA grown on solid media containing glucose or glycerol. **a** Colony color intensity of BJ5465.sfp.bpsA spotted on plates containing rich media and 2% glucose or the non-fermentable carbon source glycerol after 3 days and 7 days of growth. Colony color intensities are quantified using the Fiji image processing package distribution of ImageJ [45] and are normalized to highest detected colony intensity after brightness adjustment of the background. Error bars represent the standard deviation of 3 replicates. Representative colonies are shown in the panel below the graph. Pictures of the full plates can be found in Additional file 1: Figure S2. **b** Bright field microscopy of cells grown on the non-fermentable carbon source glycerol after 3 days and 7 days, %blue represents the percentage of pigment producing cells of 500 cells counted for each condition, ×63 magnification, scale bar = 10 µm

Thus, we hypothesized that efficient formation of indigoidine as a product of the TCA cycle takes place predominantly during the respiratory metabolic state and not during fermentative growth. To test this hypothesis, BJ5465.sfp.bpsA was grown on solid rich media containing either 2% glucose or 2% glycerol as sole carbon sources and pigment formation was monitored. When grown on medium containing glycerol, visible blue pigmentation coincided with visible colony formation after 3 days of incubation at 37 °C and increased in intensity to reach maximum pigmentation after additional 4 days (Fig. 2a). Using glucose as a carbon source caused a delay in visible pigmentation but increased growth rate of the colonies as compared to glycerol. Because glycerol is a non-fermentable carbon source, cells are required to shift into respiratory metabolic state, which leads to a decrease in growth rate but an increased flux through the TCA cycle. Furthermore, blue pigment production was absent in spontaneous petite mutants grown on medium containing 2% glucose (Additional file 1: Figure S1), indicating the requirement of functional mitochondria for indigoidine formation. Petite mutants form small colonies on fermentable carbon sources and are unable to grow on non-fermentable carbon sources due to absent or dysfunctional mitochondria and thus TCA cycle deficiency [26]. Therefore, these observations are consistent with our hypothesis that efficient production of indigoidine occurs during respiratory growth.

To determine if the increase in pigmentation for cells grown on rich medium containing glycerol as sole carbon source originates from an increase of number of cells producing the blue pigment or from an enhanced production per cell, we performed light microscopy of cells grown on glycerol. We found the cause for increased colorization of the colonies predominantly to be a result of an increase in the number of pigment-producing cells (Fig. 2b).

In addition to the type of sugar used as a carbon source, the concentration of sugar in the medium has a strong impact on the energy metabolism of *S. cerevisiae.* In aerobic conditions, *S. cerevisiae* metabolizes different carbon sources via different metabolic pathways, namely fermentative, mixed respiro-fermentative or purely respiratory for non-fermentable carbon sources [8, 14, 27]. To study the effect of increasing sugar concentration of differentially metabolized carbon sources on the production of indigoidine, we cultivated BJ5465.sfp.bpsA in liquid media containing either glucose, sucrose, galactose or glycerol ranging in concentrations from 1 to 5%.

**Fig. 3:**
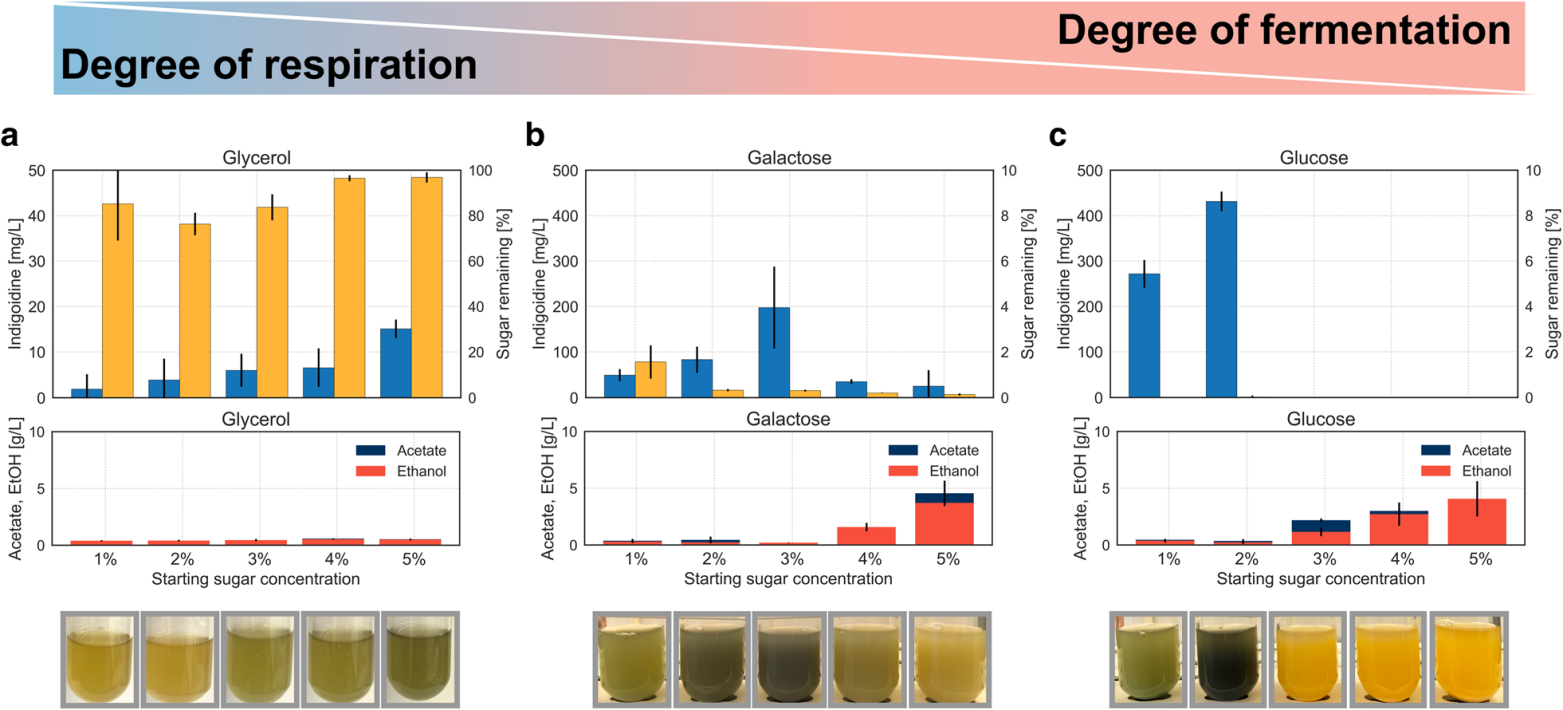
Phenotype and Titer of BJ5465.sfp.bpsA grown in different carbon sources for 3 days. BJ5465.sfp.bpsA was grown in rich media containing either glycerol (**a**), galactose (**b**) or glucose (**c**) ranging in concentrations from 1 to 5% as sole carbon source for 3 days. The carbon sources are utilized via different metabolic pathways in *S. cerevisiae*, namely respiratory for glycerol, mixed respiro-fermentative for galactose and fermentative for glucose. Top: quantification of indigoidine produced (blue bars) and remaining sugar in percentage (yellow bars) after 3 days of cultivation. Note difference in scale for indigoidine titer in glycerol compared to galactose and glucose. Middle: quantification of ethanol (red bars), acetate (dark blue bars), and indigoidine (blue bars). Bottom: representative photographs of respective liquid cultures after 3 days of cultivation. Error bars represent 95% CI (n = 4)

We expected production of indigoidine to occur when cells enter respiratory growth upon activation of the TCA cycle. Thus, we hypothesized that pigment production would start upon entering growth phase independent of sugar concentration for the non-fermentable carbon source glycerol and galactose. Indeed, we observed pigment formation in all concentrations of glycerol or galactose as carbon source (Fig. 3). While the quantified concentration of indigoidine via a colorimetric assay did not show a strong dependence on initial concentration of sugar present in the cultures grown in glycerol, a slight trend towards more intense coloration of the culture with increasing sugar concentration was observed (Fig. 3a). Even though *S. cerevisiae* is able to metabolize glycerol, growth remains slow when utilizing this non-fermentable sugar as sole carbon source [28], resulting in slow growth and thus low titers of indigoidine in these cultures. Further, for these cultures only negligible amounts of by-products were detected. In contrast, cultures grown in galactose showed the highest indigoidine production after 3 days with a starting concentration of 3% galactose, decreasing with increasing deviation from this concentration (Fig. 3b). Even though most of the sugar was already consumed after 3 days as quantified by HPLC analysis, indigoidine production increased further with starting concentrations of 2–5% galactose but remained stable for 1% after 5 days (Additional file 1: Figure S3). By-product formation was solely detected in cultures with higher starting galactose concentrations of 4% and 5%, indicating the occurrence of carbon catabolite repression as previously described by Gancedo et al. [7]. The results obtained for cultures grown in glycerol and galactose are consistent with ^13^C flux studies, showing increased flux to the precursor alpha-ketoglutarate under purely respiratory or respiro-fermentative growth [10].

For sucrose and glucose, pigment production was observed at high titers for low starting sugar concentrations of 1% and 2% but was absent at 4% and 5% initial sugar concentrations, while the by-products ethanol and acetate were detected in increasing amounts with increasing starting sugar concentrations (Fig. 3c; sucrose in Additional file 1: Figure S3).

We hypothesized that the lack of pigment production at higher starting concentrations of these fermentable carbon sources after 3 days could be caused by remaining sugar. Unconsumed sugar present in the medium at sufficient concentrations could cause the cells to remain in the fermentative state, inhibiting flux through the TCA cycle and thus preventing indigoidine production. To test this hypothesis, we performed sugar and by-product quantification using HPLC. This analysis revealed that 99% of the sugar is consumed independent of starting glucose concentrations (Fig. 3c, middle), rendering excess sugar as cause for the absence of pigment production unlikely. This conclusion is supported by the observation that pigment production remained absent in cultures of 4% or 5% glucose or sucrose concentration even after additional 48 h of cultivation (Additional file 1: Figure S3). The absence of pigment production in cultures with high initial sugar concentrations could originate from nitrogen limitations of these cultures at later stages of their growth. This conclusion is in agreement with results from observations made by Brown and Johnson [27] when analyzing the effect of sugar concentrations on cell yield and metabolites of *S. cerevisiae* cultures.

**Fig. 4:**
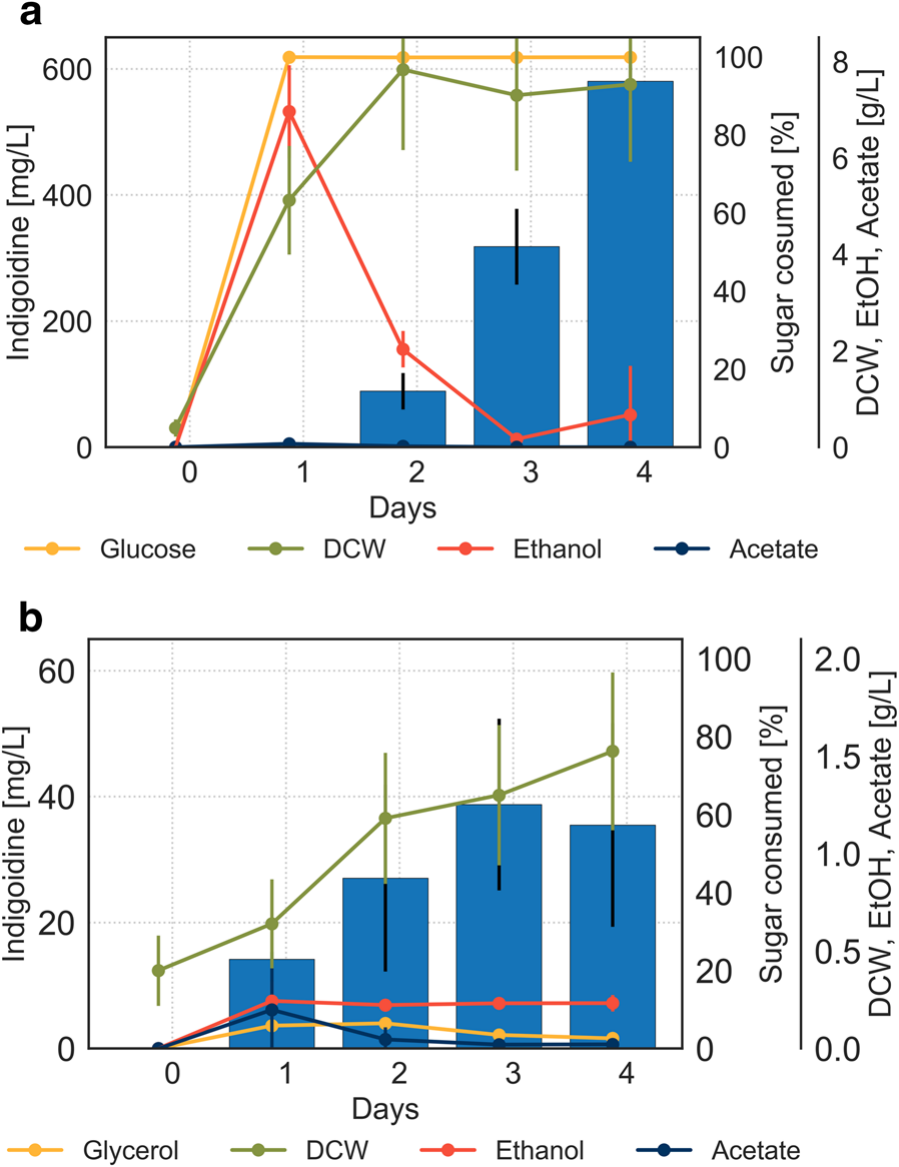
Cultivation profile of BJ5465.sfp.bpsA in different carbon sources. Concentrations of indigoidine (blue bars), consumed sugar (yellow line), dry cell weight (DCW, green line) and the by-products ethanol (red line) and acetate (dark blue line) are plotted against time for cells grown in **a** glucose and **b** glycerol. Error bars represent 95% CI (n = 4), note difference in scale between **a** and **b**

To gain a detailed understanding of the production profile, we captured the dynamics of metabolite abundances (carbon source, ethanol, acetic acid) and quantified pigment production over the course of 4 days. As expected, the carbon consumption profile of BJ5465.sfp.bpsA grown in medium containing glucose resembles a typical profile for aerobic diauxic growth by *S. cerevisiae* [29]. In the first 24 h of cultivation, glucose was fully consumed by fermentative metabolism resulting in the production of 6.96 ∓ 0.85 g/L ethanol, 0.07 ∓ 0.01 g/L acetate and biomass accumulation of 5.13 ∓ 0.78 g/L (Fig. 4a). In a subsequent respiratory metabolic growth phase, the non-fermentable carbon source ethanol was consumed leading to slower biomass formation. The shift from glucose consumption to ethanol consumption, marks the onset of indigoidine production after 24 h. These results indicate that indigoidine production coincided with the shift from fermentative to respiratory metabolism for cells grown on glucose containing medium. Thus, we expected that growth on a non-fermentable carbon source would eliminate the delay in indigoidine production caused by an initial fermentative growth phase. Indeed, growth on glycerol resulted in prompt production of blue pigment (Fig. 4b), even though glycerol was consumed at very slow rates throughout the experiment. Ethanol and acetate were produced at negligible amounts during the entire growth phase as expected for respiratory growth. In contrast to growth on glucose, indigoidine production profile during growth on glycerol correlated with the biomass profile.

### Altering metabolic states through controlled carbon availability in bioreactors can enhance indigoidine production

Advanced process control available in bioreactors can be used to influence microbial growth and product generation through controlled culture environments. Our previous experiments were performed in tubes and shake flasks in batch-mode, where no additional substrate was added after the start of the cultivations. In these batch fermentations, substrate depletion affected the metabolic state of the cultures. To maintain a specific metabolic state over an extended period of time, fed-batch cultivations with two different substrate feeding strategies, i.e. carbon depletion and carbon excess, were performed in 2 L bioreactors.

Carbon depletion conditions were implemented using a dissolved oxygen (DO) signal-based pulse feeding strategy. The metabolic activity of cells stalls upon depletion of total carbon in the culture. First, glucose is fully consumed, followed by the consumption of other sources of carbon such as fermentative by-products ethanol and acetate. The stall in metabolic activity leads to a reduction in oxygen demand, resulting in a sudden increase (“spike”) of dissolved oxygen levels in the culture. A “pulsed” feed of glucose was triggered upon carbon depletion events detected by a DO spike. Excess availability of carbon was achieved through semi-continuous feeding of glucose with fixed delivery of 4 g/L/h.

**Fig. 5:**
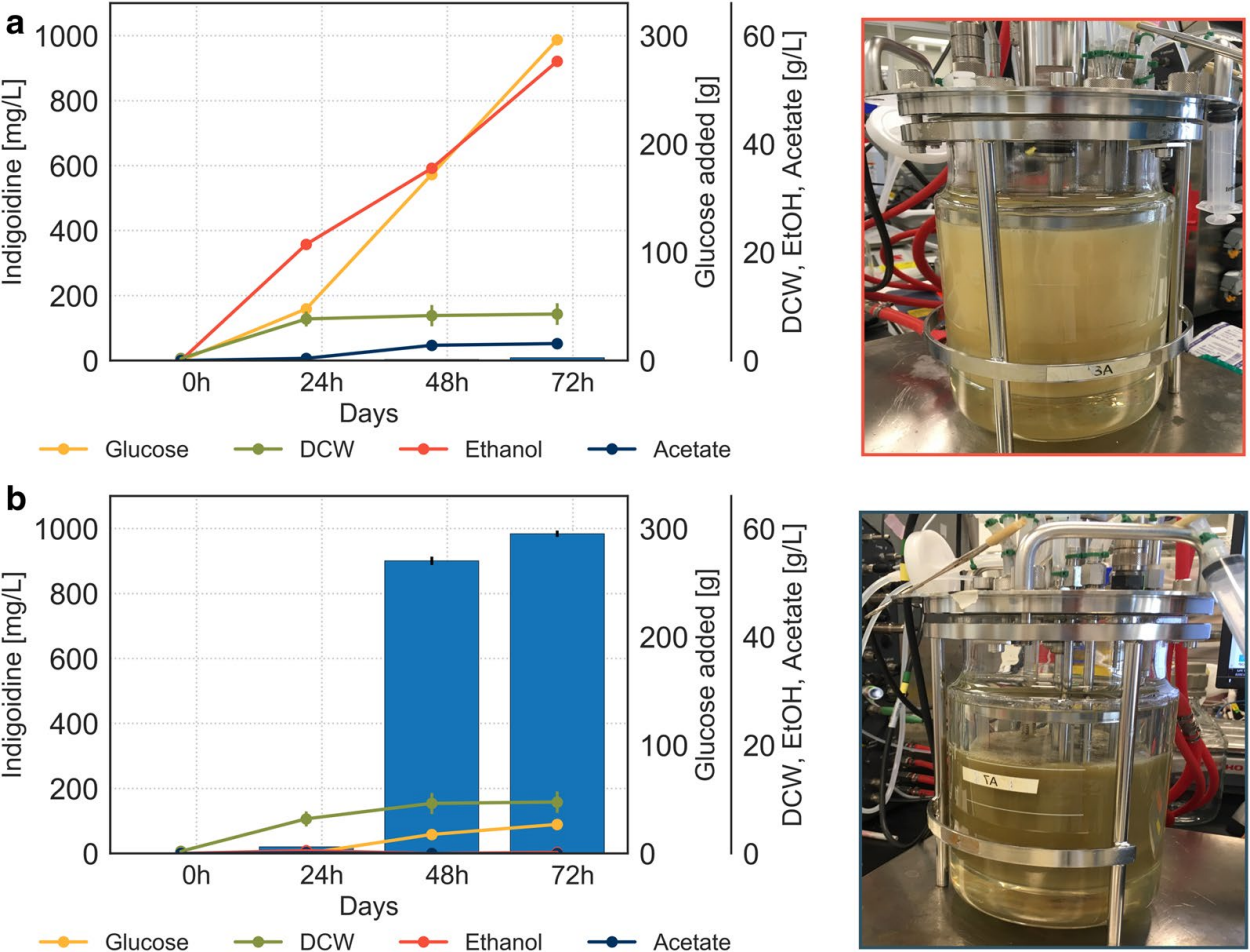
Regulated environment in 2 L bioreactor enables control over metabolic state. Fed-batch fermentation of BJ5465.sfp.bpsA with **a** excess glucose feed or **b** signal-based pulse feeding strategy resulting in glucose starvation conditions. Lines represent concentrations of total glucose fed and ethanol and acetate produced; bars represent indigoidine concentration. N = 3 technical replicates for indigoidine extraction and DCW measurements. Additional process parameters and gas analysis can be found in Additional file 1: Figures S4 and S5

We hypothesized that excess carbon conditions would promote fermentative metabolism while carbon depletion would enable respiratory metabolism. Indeed, excess conditions resulted in accumulation of the by-products ethanol and acetate reaching final concentrations of 55.3 g/L and 3.1 g/L, respectively (Fig. 5a). No significant production of the pigment was observed. These observations agree with our hypothesis that fermentative metabolic state and thus the inactivity of the TCA cycle impedes efficient pigment formation.

In contrast, depletion conditions resulted in a high production titer of indigoidine, reaching 980 mg/L at the end of the fermentation while accumulating only negligible amounts of ethanol and acetate throughout (Fig. 5b). Interestingly, excess availability of glucose did not have a significant effect on biomass formation as determined by dry cell weight. These results indicate that the growth parameters selected for glucose starvation conditions imposed a predominantly respiratory metabolic state leading to the activation of the TCA cycle and production of indigoidine.

## Conclusion

Our findings demonstrate that the metabolic state of the cell is critical for the efficiency of a biosynthetic pathway. We showed that production of the NRPS catalyzed blue pigment indigoidine, a product of the TCA cycle, is linked to the respiratory metabolic state in *S. cerevisiae*. Important cultivation parameters, known to affect the metabolic state of *S. cerevisiae,* shape the indigoidine production profile regarding timing and titer. In the case of non-fermentable carbon sources that are consumed via respiratory metabolism, pigment production occurs at the same time as biomass formation (e.g. DCW). In contrast, growth on glucose results in a delay of indigoidine production until after the glucose is consumed.

Our results are consistent with ^13^C metabolic flux studies that report redirection of flux towards the TCA cycle during respiratory metabolism compared to fermentation [10, 12]. While it is known that several native pathways undergo a redirection of flux accompanying the shift from fermentative to respiratory metabolism in *S. cerevisiae* [11, 18, 30], these aspects are rarely considered during initial strain engineering or demonstration of production. However, as shown in this study, changes in the metabolic flux profile and precursor pools do have major implications for the productivity of the host cell. In this context, the TCA cycle is specifically important as it is not only the main pathway for generation of reducing equivalents, but also produces important intermediates and precursors for biosynthetic products such as amino acids from the aspartate and alpha-Ketoglutarate families [31, 32] and short-chain dicarboxylic acids such as succinate [33, 34]. In addition to the TCA cycle, metabolic flux of other pathways, commonly employed in metabolic engineering, like the pentose phosphate pathway and the glyoxylate cycle have also been shown to be affected by metabolic redirections [10, 12].

The dependence of metabolic state and production efficiency of biosynthetic pathways becomes increasingly important when transitioning into industrial scale production or fed-batch mode. In these conditions, insufficient mixing commonly leads to heterogeneity in substrate and oxygen distribution [3]. Indeed, Fu et al. [35] reported a prominent difference in glucose catabolism in *S. cerevisiae* in response to transitioning from laboratory (10 L) to industrial scale (10,000 L), and detected a loss of TCA cycle intermediates through secretion relating to mitochondrial dysfunction at industrial as compared to laboratory scale. Our findings underscore the need for identification of production strains that maintain robust performance in the presence of large concentration gradients over the course of a production process with in a bioreactor. In addition to constructing strains with the desired biosynthetic pathway, several strain attributes and host selection criteria need to be considered a priori, for a given final product, to effectively develop engineered microbes well-suited for large-scale aerobic cultivation [36]. Given the growing potential of metabolic engineering tools available, a solution to this problem can be seen in “rewiring” the central carbon metabolism to increase the energy efficiency of the production pathway of a given production strain and thereby reducing the oxygen demand, for example to increase the efficiency of acetyl CoA-based isoprenoid production in *S. cerevisiae* [37].

To our knowledge, this is the first report of a high-titer production of the non-ribosomal peptide indigoidine in a fungal host, achieving 980 mg/L indigoidine at a 2 L bioreactor scale. We demonstrated that indigoidine formation is linked the respiratory metabolic state in *S. cerevisiae* and maintenance of the required metabolic state was critical for enhancing its production levels at higher scales [38, 39]. Our study illustrates that a better understanding of the metabolic states involved in heterologous production in the respective production environment is imperative for a reliable outcome in strain performance and has to be taken into consideration during strain engineering. In addition to contributing to understanding the importance of the metabolic state of the production host for optimal performance in bioprocesses, our system may be used as a control for metabolic state during strain and process development.

## Materials and methods

### Strain construction

All *S. cerevisiae* strains used in this study are derived from the protease deficient strain BJ5465: MATa *ura*3-52 *trp*1 *leu*2-Δ1 *his*3-δ200 *pep*4::*HIS*3 *prb*1-δ1.6R *can*1 *GAL* (ATCC). All strains and strain information have been deposited in the public instance of the JBEI Registry [40] (https://public-registry.jbei.org/folders/386) and are physically available from the authors upon request.

To create the strain BJ5465.sfp.bpsA, *sfp* was integrated into the yeast chromosomal δ-sequences [41]. The *bpsA* gene was codon-optimized for expression in *S. cerevisiae* (Genscript, Piscataway NJ) and genomically integrated into locus ARS1014a under control of the *TDH3* promoter and *ADH1* terminator using a previously reported, cloning free Cas9 toolkit [42]. Transformations were performed using the conventional lithium acetate method [43] using 200 ng pCut_1014a and 500 ng of linear Donor DNA with 500 bp homology to the integration locus ARS1014a.

*E. coli* strain Bap1 [44] was transformed with a E5C plasmid encoding bpsA codon-optimized for expression *S. cerevisiae* (Additional file 1: Figure S6) and used as a host to establish indigoidine production and prepare a standard curve for quantification of pigment production (Additional file 1: Figure S7).

### Media and cultivation conditions

Overnight cultures of *S. cerevisiae* were grown in 5 mL standard rich Glucose medium (YPD, 1% (w/v) Bacto yeast extract, 2% (w/v) Bacto peptone, 2% (w/v) Dextrose) at 30 °C, shaking at 200 rpm. Production cultures were inoculated to an OD_600_ of 0.05 in rich medium [YP, 1% (w/v) Bacto yeast extract, 2% (w/v) Bacto peptone and 2% (w/v) Sugar], unless stated otherwise and grown at 30 °C at 200 rpm. All productions were carried out in quadruplets.

### Imaging and color intensity quantification

Pictures of plates and culture tubes were taken with a 12-megapixel camera. The means of color intensity of three colonies was quantified using the Fiji image processing package distribution of ImageJ [45]. For this analysis, the colorization of the plates was adjusted to match, based on background color. The analysis was carried out for three different colonies from three technical replicates each.

For brightfield microscopy, 1 μL of cells from liquid culture or an equivalent of 1 μL from colonies grown on agar plates were imaged for blue pigment production studies using a Leica-DM4000B microscope equipped with a Hamamatsu Digital Camera C4742-95 and a Micropublisher 5.0 RTV Camera with a 63× or 100× objective and processed using Leica software (Leica Application Suite X, LAS X). To determine the ratio of pigment producing cells of cells in a population, 500 cells each were counted and categorized from microscopy pictures.

### Indigoidine extraction

Purification of indigoidine was performed using a modified protocol from Yu et al. [46]. Briefly, 1 mL of culture was centrifuged at 21,000×*g* for 3 min and the supernatant removed. To lyse the cells and extract indigoidine, 100 μL of acid washed beads (625 nm) and 1 mL DMSO + 2% Tween^®^ 20 were added to the cell pellet and vortexed twice for 1 min using Mini-Beadbeater-96 (Biospec, Bartlesville OK) at 3600 rpm. After centrifugation at 21,000×*g* for 3 min, the indigoidine concentration was determined by measuring the OD_612_ of the supernatant using a BioTek Synergy 4 plate reader (Biotek, Winooski VT), preheated to 25 °C and applying a standard curve.

### Preparation of indigoidine standard curve

The *E. coli* strain Bap1 E5C.bpsA was grown overnight at 37 °C in 5 mL LB medium (Beckton Dickinson, NJ, USA; Cat. No. 244610) supplemented with 25 µg/mL Chloramphenicol and back diluted to OD_600_ of 0.1 in 10 mL LB Chloramphenicol the next morning. The strain was cultivated at 37 °C shaking at 200 rpm to reach OD_600_ of 0.4, induced with 1 mM isopropyl-1-thio-β-d-galactopyranoside (IPTG, Sigma-Aldrich, St. Louis MO) and further cultivated at 30 °C, 200 rpm for 24 h before harvesting the cell pellet by centrifugation 10,000×*g* for 5 min. To lyse the cells and extract indigoidine, 100 μL of acid washed beads (625 nm) and 1 mL DMSO + 2% Tween^®^ 20 were added to the cell pellet and vortexed twice for 1 min using Mini-Beadbeater-96 (Biospec, Bartlesville OK) at 3600 rpm. The mixture was centrifuged, and the supernatant was dried in vacuo. To obtain pure indigoidine, the resulting pellet was washed twice each with 1 mL water, 1 mL EtOAc, 1 mL MeOH and 1 mL Hexane and dried again in vacuo. Afterwards, 0.64 mg of dried indigoidine was dissolved in 1 mL DMSO. This solution was further serially diluted into six different concentrations (0.01, 0.02, 0.04, 0.08, 0,16, 0.32 mg/mL) and measured for OD_612_ values using a BioTek Synergy 4 plate reader, preheated to 25 °C. The standard curve was established by the linear relationship between the absorbance and concentration according to Kuhn et al. [47] and is shown in Additional file 1: Figure S7.

### Quantification of sugar, ethanol and acetate

Sugar and by-product concentrations were quantified on a 1200 series HPLC (Agilent Technologies) equipped with an Aminex H column (Bio-Rad, Hercules CA). Samples were filtered through 0.45 μm filters (VWR) to remove cells, and 5 μL of each sample was injected onto the column, preheated to 50  °C. The column was eluted with 4 mM H_2_SO_4_ at a flow rate of 600 μL/min for 25 min. Sugars and metabolites were monitored by a refractive index detector, and concentrations were calculated by peak area comparison to known standards.

### Fed-batch experiments at 2 L bioreactor scale

Fed-batch experiments were performed using 2 L Sartorius BIOSTAT B^®^ fermentation system (Sartorius AG., Goettingen, Germany), each agitated with two Rushton impellers, with an initial working volume of 1.5 L YP1%D [1% (w/v) Bacto yeast extract, 2% (w/v) Bacto peptone, 1% (w/v) dextrose] and 50 mL seed culture.

The bioreactor cultivations were inoculated at pH 6.6. The pH was not controlled throughout the course of the experiment. A 600 g/L glucose solution was used as carbon feed. DO was controlled at 30% saturation by varying agitation from 400 to 600 rpm (cascade mode to control DO in batch phase and fed-batch phase unless stated otherwise), at an aeration rate of 1.5 LPM (1 VVM). Fermentation temperature was held constant at 30 °C.

Process values were monitored and recorded using the integrated Sartorius software (BioPAT MFCS/win). Feeding parameters were implemented using customized LabVIEW Virtual Instruments (National Instruments, Austin, TX). Exhaust gas oxygen and carbon dioxide compositions were monitored and recorded using BlueSens offgas analyzers (BlueInOne Cell, BlueSens gas sensor GmbH, Herten, Germany).

Glucose starvation conditions were achieved by utilizing a DO-based pulse feeding strategy in which glucose was added on demand upon carbon exhaustion. The pulse parameters for the pulse-feed experiments were as follows: Pulse trigger condition were optimized after 17 h of the cultivation to increase the number of starvation events by reducing the amount of glucose fed per pulse (3 g per pulse to 0.6 g per pulse after 17 h). The pulse trigger conditions were as follows: ΔDO = 20%; flow rate; 0.167 mL/min; pulse duration, 30 min (first 17 h of feed phase) and 6 min (until end of fermentation).

Glucose excess conditions were achieved by utilizing a fixed rate pulse feeding strategy which aimed at restoring the initial batch glucose concentration of 10 g/L, followed by periodic glucose pulse additions administering a fixed pulse dosage of 10 mL of glucose feed solution or 6 g glucose per hour (4 g/L/h). It is important to note that we did not observe glucose accumulation larger than 1 g/L in the excess feeding strategy.

## Additional file

**Additional file 1: Figure S1.** Phenotype of BJ5465.sfp.bpsA producing Indigoidine streaked on YPD plates. Overnight cultures of BJ5465.sfp.bpsA grown in 3 mL YPD media were streaked onto YPD plates and incubated at 30 °C. Indigoidine production was monitored over the course of 10 days. Red boxes mark spontaneous white mutants forming small colonies. Because these mutants did not grow on glycerol as sole carbon source (data not shown), we conclude that these mutants lack functional mitochondria and can be characterized as petites. **Figure S2.** Phenotype of BJ5465.sfp.bpsA spotted on solid media containing either glucose (YPD or glycerol (YPG). Overnight cultures of BJ5465.sfp.bpsA grown in 3 mL YPD media were spotted in twofold serial dilution onto YPD and YPG plates and incubated at 30 °C. Indigoidine production was monitored over the course of 7 days. **Figure S3.** Phenotype and Titer of BJ5465.sfp.bpsA grown in different carbon sources. BJ5465.sfp.bpsA was grown in rich media containing either glycerol, galactose, sucrose or glucose ranging in concentrations from 1 to 5% as the sole carbon source for 5 days. Indigoidine production was quantified after 3 days (dark blue bars) and 5 days (light blue bars). The carbon sources are utilized via different metabolic pathways in *S. cerevisiae*, namely respiratory for glycerol, mixed respiro-fermentative for galactose and fermentative for glucose. Error bars represent standard deviation (n = 3–4). **Figure S4.** Time profiles of dissolved Oxygen and Oxygen Uptake Rate during glucose starvation conditions at 2 L bioreactor scale. Glucose starvation conditions were realized using a DO signal-based pulse feeding strategy adding 0.4 g glucose per liter on demand upon carbon source depletion. Feed start after 24 h. Spikes in dissolved Oxygen were used to trigger feed pulses. Dissolved Oxygen (pO_2_) is shown in blue and Oxygen Uptake Rate (OUR) is shown in red. **Figure S5.** Time profiles of dissolved Oxygen and Oxygen Uptake during excess glucose conditions at 2 L bioreactor scale. Excess glucose was achieved via a semi-continuous feeding strategy with fixed glucose delivery of 4 g glucose per liter per hour. Dissolved Oxygen (pO_2_) is shown in blue and Oxygen Uptake Rate (OUR) is shown in red. **Figure S6.** DNA sequences and constructs used in this study. **A** Sequence of *Streptomyces lavendulae bpsA* codon optimized for expression in *S. cerevisiae*. **B** The codon-optimized version was cloned into an E5C plasmid backbone under the control of an IPTG inducible lacUV5 promoter for production of Indigoidine in *E. coli.* **Figure S7.** Standard curve of Indigoidine absorbance at 612 nm in DMSO. Absorbance values were obtained for serial dilutions of purified Indigoidine in DMSO. The equation for the trendline is y = 0.739x − 0.0721, R^2^ = 0.9948.
