# Correction to: Production efficiency of the bacterial non-ribosomal peptide indigoidine relies on the respiratory metabolic state in *S. cerevisiae*

**DOI:** 10.1186/s12934-019-1262-2

**Published:** 2019-12-29

**Authors:** Maren Wehrs, Jan-Philip Prahl, Jadie Moon, Yuchen Li, Deepti Tanjore, Jay D. Keasling, Todd Pray, Aindrila Mukhopadhyay

**Affiliations:** 1 Biological Systems and Engineering Division, Lawrence Berkeley National Laboratory, Berkeley, CA 94720 USA; 2 Institut für Genetik, Technische Universität Braunschweig, Brunswick, Germany; 3 Joint BioEnergy Institute, Lawrence Berkeley National Laboratory, Emeryville, CA 94608 USA; 4 Advanced Biofuels and Bioproducts Process Development Unit, Lawrence Berkeley National Laboratory, Emeryville, CA 94608 USA; 5 Department of Plant and Microbial Biology, University of California, Berkeley, CA 94720 USA; 6 Department of Bioengineering, University of California, Berkeley, CA 94720 USA; 7 Department of Chemical and Biomolecular Engineering, University of California, Berkeley, CA 94720 USA; 8 The Novo Nordisk Foundation Center for Biosustainability, Technical University of Denmark, Kongens Lyngby, Denmark; 9 Synthetic Biochemistry Center, Institute for Synthetic Biology, Shenzhen Institutes for Advanced Technologies, Shenzhen, China; 10 Environmental Genomics and Systems Biology Division, Lawrence Berkeley National Laboratory, Berkeley, CA 94720 USA

## Correction to: Microb Cell Fact (2018) 17:193 10.1186/s12934-018-1045-1

Following publication of the original article [1], the authors have noted that the standard curve in Additional file 1: Figure S7 is incorrect.

The authors have since corrected the standard curve for Additional file 1: Figure S7 and recalculated the Titers (as g L^−1^) of the final product Indigoidine.

Please note that by using the corrected standard curve, the titer values in the following figures are altered: Figs. 3, 4, and 5.

**Fig. 3:**
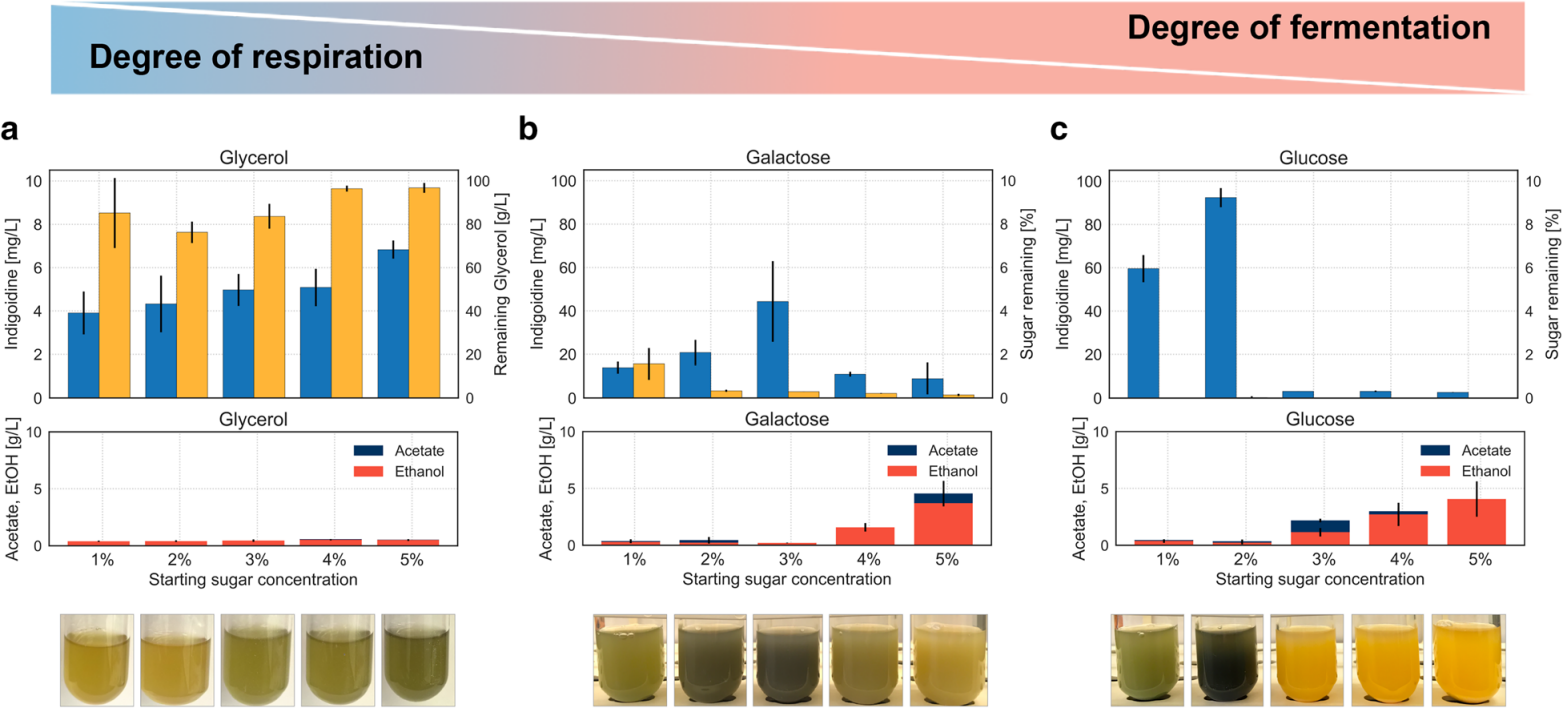
Phenotype and Titer of BJ5465.sfp.bpsA grown in different carbon sources for 3 days. BJ5465.sfp.bpsA was grown in rich media containing either glycerol (**a**), galactose (**b**) or glucose (**c**) ranging in concentrations from 1 to 5% as sole carbon source for 3 days. The carbon sources are utilized via different metabolic pathways in *S. cerevisiae*, namely respiratory for glycerol, mixed respiro-fermentative for galactose and fermentative for glucose. Top: Quantification of indigoidine produced (blue bars) and remaining sugar in percentage (yellow bars) after 3 days of cultivation. Note difference in scale for indigoidine titer in glycerol compared to galactose and glucose. Middle: Quantification of ethanol (red bars), acetate (dark blue bars), and indigoidine (blue bars). Bottom: Representative photographs of respective liquid cultures after 3 days of cultivation. Error bars represent 95% CI (n = 4)

**Fig. 4:**
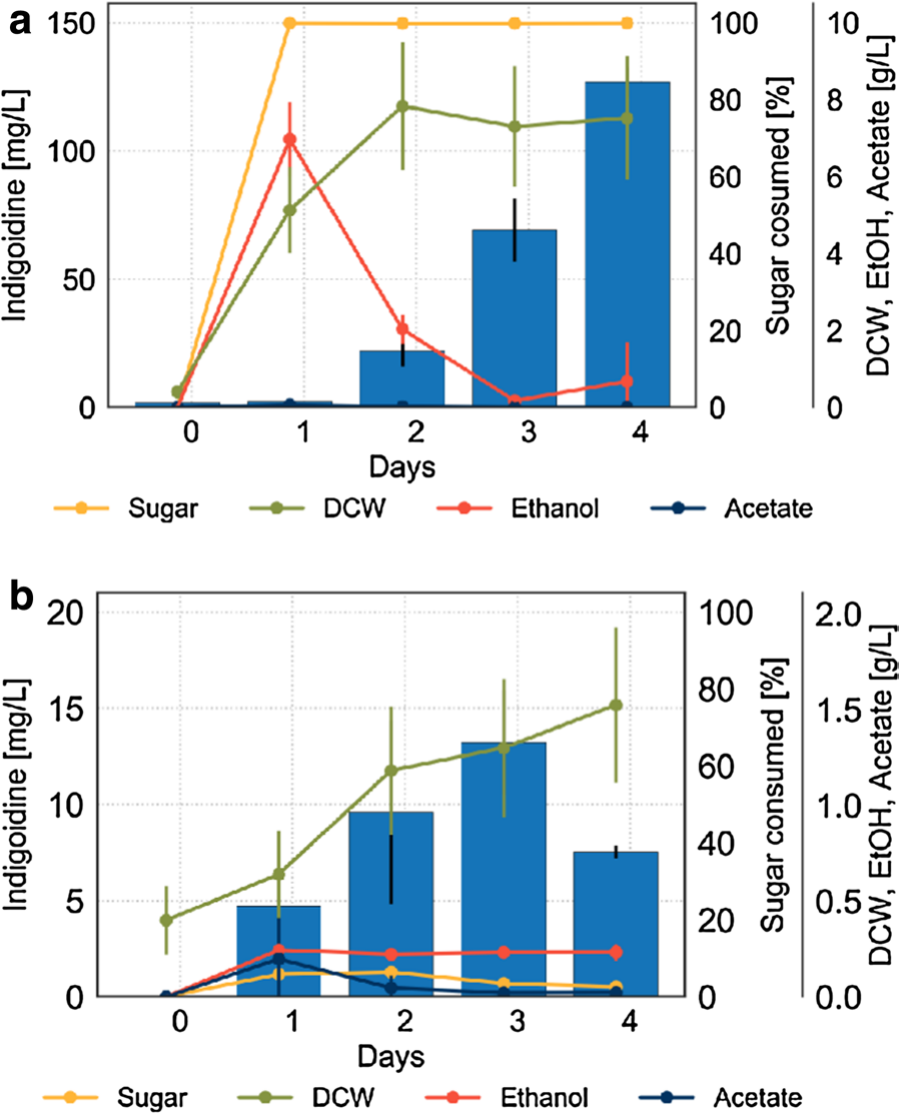
Cultivation profile of BJ5465.sfp.bpsA in different carbon sources. Concentrations of indigoidine (blue bars), consumed sugar (yellow line), dry cell weight (DCW, green line) and the by-products ethanol (red line) and acetate (dark blue line) are plotted against time for cells grown in **a** glucose and **b** glycerol. Error bars represent 95% CI (n = 4), note difference in scale between **a** and **b**

**Fig. 5:**
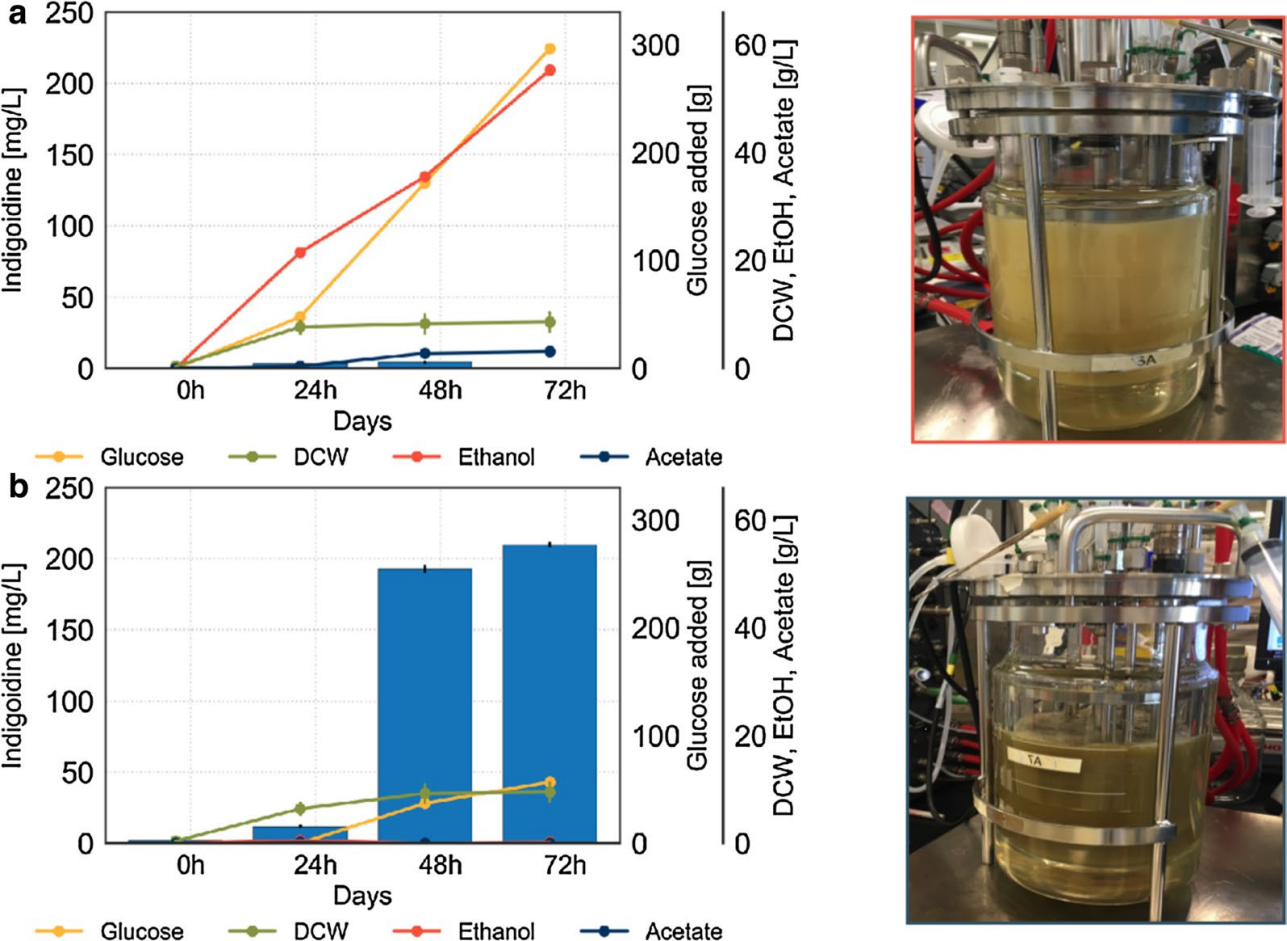
Regulated environment in 2 L bioreactor enables control over metabolic state. Fed-batch fermentation of BJ5465.sfp.bpsA with **a** excess glucose feed or **b** signal-based pulse feeding strategy resulting in glucose starvation conditions. Lines represent concentrations of total glucose fed and ethanol and acetate produced; bars represent indigoidine concentration. N = 3 technical replicates for indigoidine extraction and DCW measurements. Additional process parameters and gas analysis can be found in Additional file 1: Figures S4 and S5

However, none of the raw measurements have changed, the Figure captions are not affected and the conclusions remain unchanged.

The corrections are provided by this article (please find detailed below).

Statements in the manuscript that refer to the numerical titer values are corrected as follows:

- *In the abstract*, the corrected manuscript should state:
- ...reaching a maximum titer of 209.9 mg/L...
- *In results on page 6,* the corrected manuscript should state:
- ...reaching 209.9 mg/L...
- *In conclusions on page 7,* the corrected manuscript should state:
- ...achieving 209.9 mg/L...

## Supplementary information

**Additional file 1: Figure S7.** Standard curve of Indigoidine absorbance at 612 nm in DMSO. Absorbance values were obtained for serial dilutions of purified Indigoidine in DMSO. The equation for the trendline is: y = 0.152x − 0.111 R^2^ = 0.9986. Indigoidine was purified from microbial cultures per Yu et al. (https://doi.org/10.1007/s10295-012-1207-9). Figure shows one representative plot (of three), each with measurements in triplicate.

## References

[1] Wehrs M, Prahl J-P, Moon J, Li Y, Tanjore D, Keasling JD, Pray T, Mukhopadhyay A (2018). Production efficiency of the bacterial non-ribosomal peptide indigoidine relies on the respiratory metabolic state in *S. cerevisiae*. Microb Cell Fact.

