# Supplementary material for: Production efficiency of the bacterial non-ribosomal peptide indigoidine relies on the respiratory metabolic state in S. cerevisiae
Source: Microb Cell Fact. 2018 Dec 13;17:193. doi: 10.1186/s12934-018-1045-1 (PMC6293659; doi:10.1186/s12934-018-1045-1)
Supplement: Supplementary file 1 — Additional file 1: Figure S1. Phenotype of BJ5465.sfp.bpsA producing Indigoidine streaked on YPD plates. Overnight cultures of BJ5465.sfp.bpsA grown in 3 mL YPD media were streaked onto YPD plates and incubated at 30 °C. Indigoidine production was monitored over the course of 10 days. Red boxes mark spontaneous white mutants forming small colonies. Because these mutants did not grow on glycerol as sole carbon source (data not shown), we conclude that these mutants lack functional mitochondria and can be characterized as petites. Figure S2. Phenotype of BJ5465.sfp.bpsA spotted on solid media containing either glucose (YPD or glycerol (YPG). Overnight cultures of BJ5465.sfp.bpsA grown in 3 mL YPD media were spotted in twofold serial dilution onto YPD and YPG plates and incubated at 30 °C. Indigoidine production was monitored over the course of 7 days. Figure S3. Phenotype and Titer of BJ5465.sfp.bpsA grown in different carbon sources. BJ5465.sfp.bpsA was grown in rich media containing either glycerol, galactose, sucrose or glucose ranging in concentrations from 1 to 5% as the sole carbon source for 5 days. Indigoidine production was quantified after 3 days (dark blue bars) and 5 days (light blue bars). The carbon sources are utilized via different metabolic pathways in S. cerevisiae, namely respiratory for glycerol, mixed respiro-fermentative for galactose and fermentative for glucose. Error bars represent standard deviation (n = 3–4). Figure S4. Time profiles of dissolved Oxygen and Oxygen Uptake Rate during glucose starvation conditions at 2 L bioreactor scale. Glucose starvation conditions were realized using a DO signal-based pulse feeding strategy adding 0.4 g glucose per liter on demand upon carbon source depletion. Feed start after 24 h. Spikes in dissolved Oxygen were used to trigger feed pulses. Dissolved Oxygen (pO2) is shown in blue and Oxygen Uptake Rate (OUR) is shown in red. Figure S5. Time profiles of dissolved Oxygen and Oxygen Uptake during excess glu [file 12934_2018_1045_MOESM1_ESM.docx]

Additional file 1

Respiratory metabolic state drives the efficiency of a heterologous production pathway in *S. cerevisiae*

Maren Wehrs ^1,2,3^, Jan-Philip Prahl ^1,4^, Jadie Moon ^1,3^, Yuchen Li ^1,3^, Deepti Tanjore ^1,4^, Jay D. Keasling ^1,3,5,6,7,8,9^, Todd Pray ^1,4^, Aindrila Mukhopadhyay ^1,3,10*^

^1^Biological Systems and Engineering Division, Lawrence Berkeley National Laboratory, Berkeley, CA 94720, ^2^Institut für Genetik, Technische Universität Braunschweig, Braunschweig, Germany, ^3^Joint BioEnergy Institute, Lawrence Berkeley National Laboratory, Emeryville, CA 94608, ^4^ABPDU, Lawrence Berkeley National Laboratory, Emeryville, CA 94608, ^5^Department of Plant and Microbial Biology, University of California, Berkeley, CA 94720, USA, ^6^Department of Bioengineering, University of California, Berkeley, CA 94720, USA, ^7^Department of Chemical and Biomolecular Engineering, University of California, Berkeley, CA 94720, USA, ^8^The Novo Nordisk Foundation Center for Biosustainability, Technical University of Denmark, Denmark, ^9^Synthetic Biochemistry Center, Institute for Synthetic Biology, Shenzhen Institutes for Advanced Technologies, Shenzhen, China, ^10^Environmental Genomics and Systems Biology Division, Lawrence Berkeley National Laboratory, Berkeley, CA 94720

*Corresponding author

| Day 3 | Day 7 | Day 10 |
| --- | --- | --- |
| 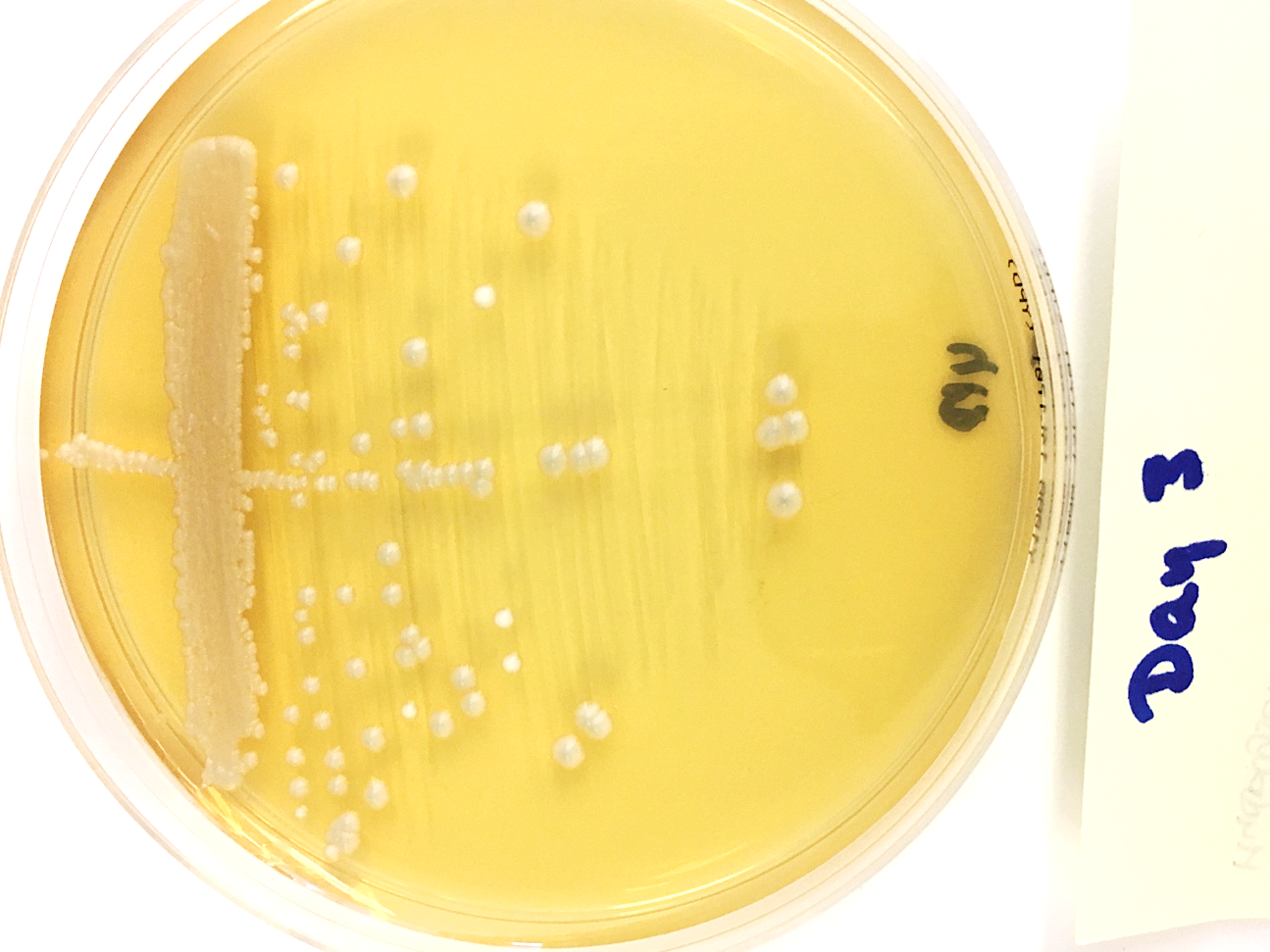 | 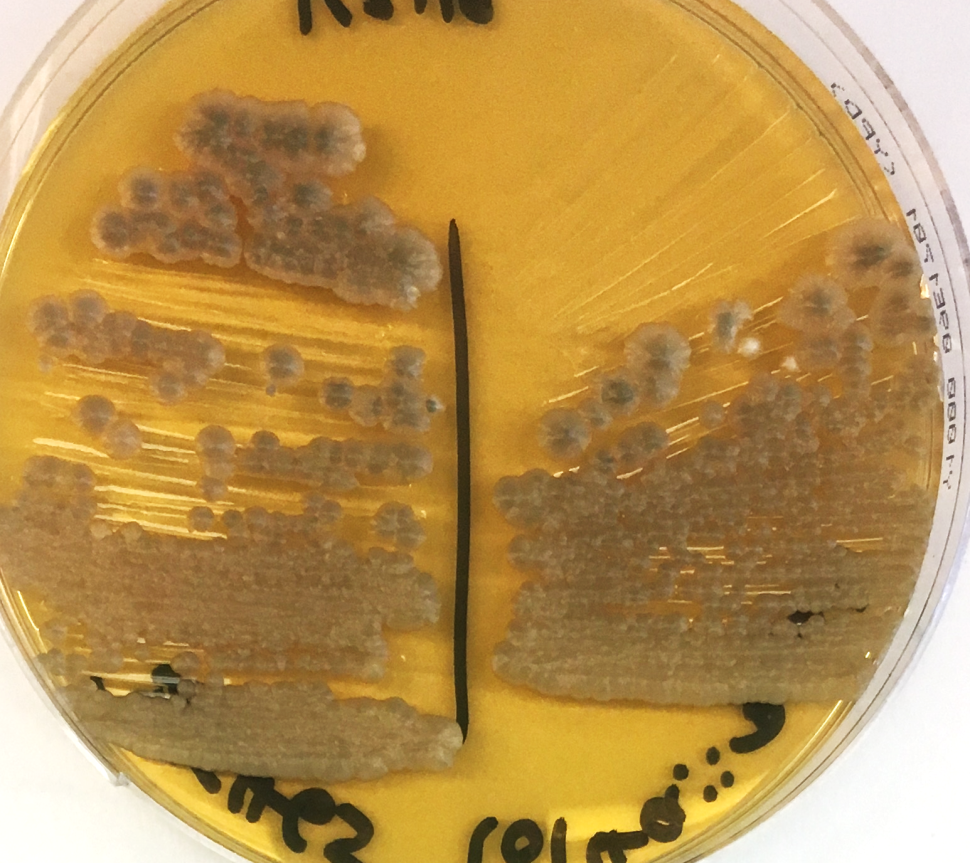 | 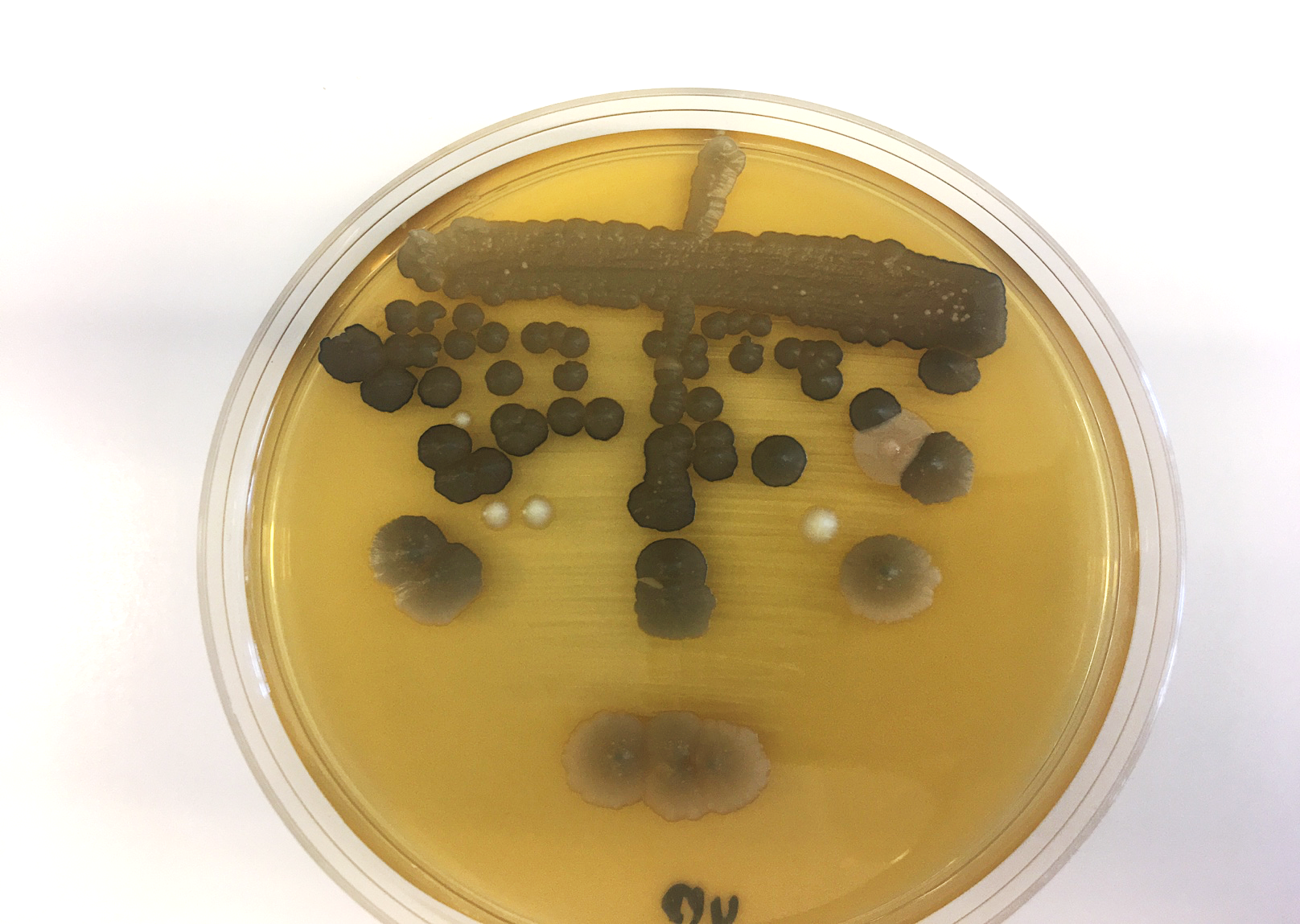 |

**Figure S1 Phenotype of BJ5465.sfp.bpsA producing Indigoidine streaked on YPD plates**

Overnight cultures of BJ5465.sfp.bpsA grown in 3 mL YPD media were streaked onto YPD plates and incubated at 30 °C. Indigoidine production was monitored over the course of 10 days. Red boxes mark spontaneous white mutants forming small colonies. Because these mutants did not grow on glycerol as sole carbon source (Data not shown), we conclude that these mutants lack functional mitochondria and can be characterized as petites.

|  | Day 3 | Day 7 |
| --- | --- | --- |
| YPD | 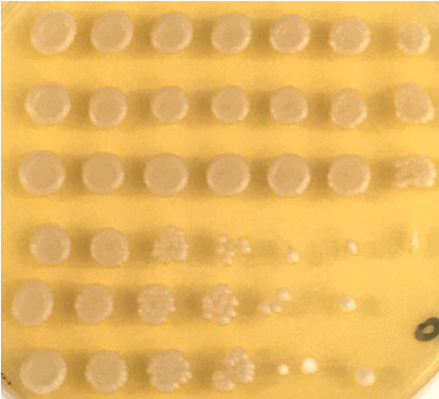 | 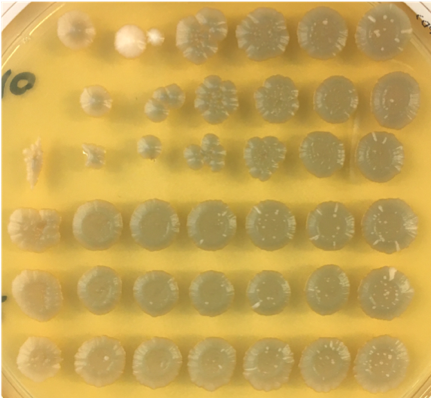 |
| YPG | 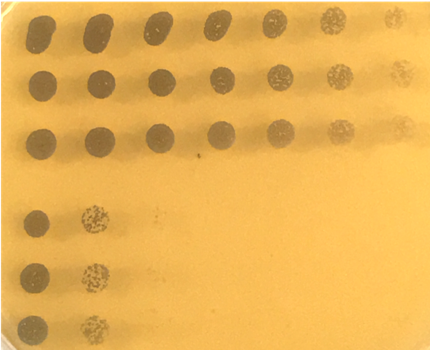 | 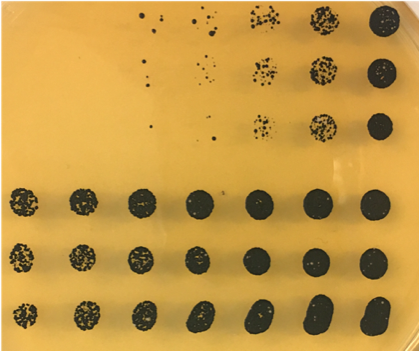 |

**Figure S2 Phenotype of BJ5465.sfp.bpsA spotted on solid media containing either glucose (YPD or glycerol (YPG)** Overnight cultures of BJ5465.sfp.bpsA grown in 3 mL YPD media were spotted in 2-fold serial dilution onto YPD and YPG plates and incubated at 30 °C. Indigoidine production was monitored over the course of 7 days.


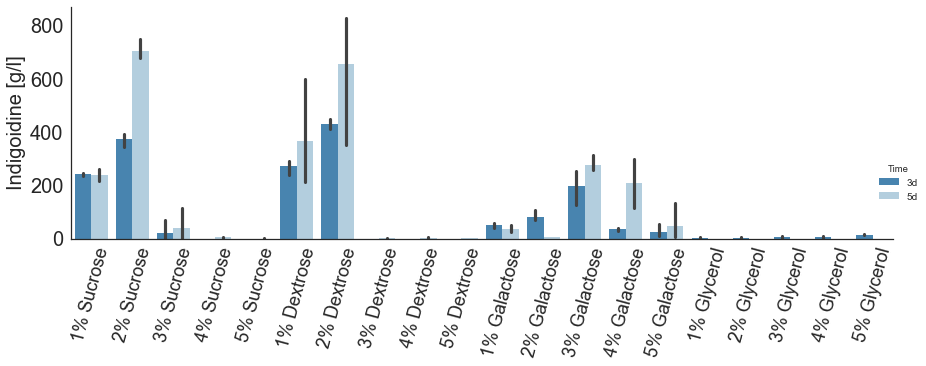


**Figure S3 Phenotype and Titer of BJ5465.sfp.bpsA grown in different carbon sources** BJ5465.sfp.bpsA was grown in rich media containing either glycerol, galactose, sucrose or glucose ranging in concentrations from 1% to 5% as the sole carbon source for five days. Indigoidine production was quantified after three days (dark blue bars) and five days (light blue bars). The carbon sources are utilized via different metabolic pathways in *S. cerevisiae*, namely respiratory for glycerol, mixed respiro-fermentative for galactose and fermentative for glucose. Error bars represent standard deviation (n = 3-4).

**
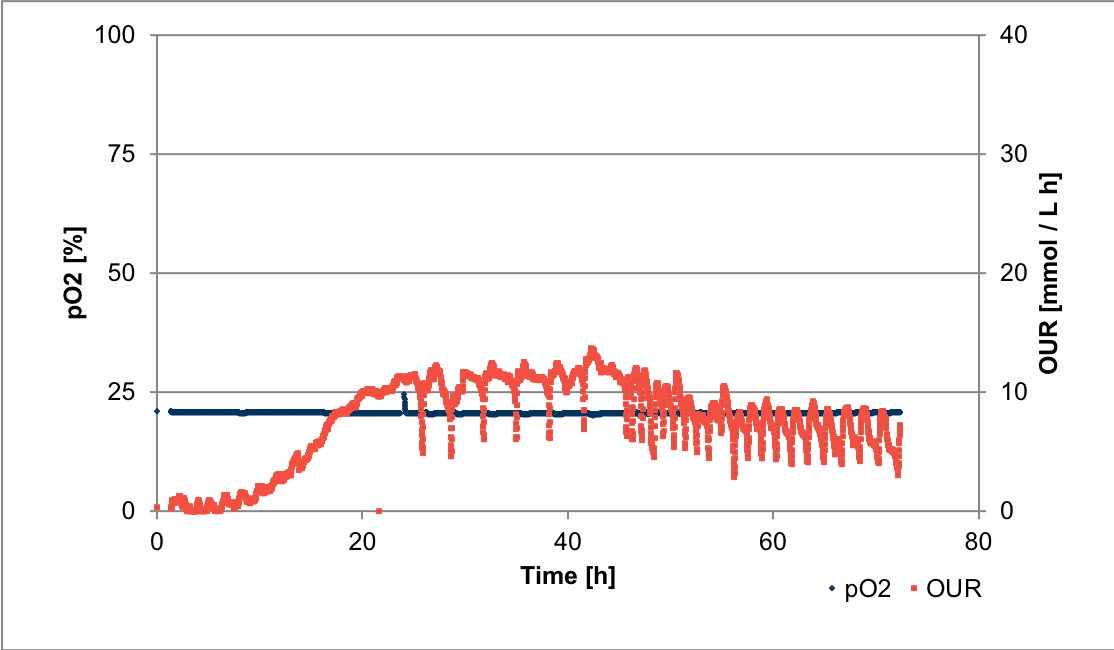
**

**Figure S4 Time profiles of dissolved Oxygen and Oxygen Uptake Rate during glucose starvation conditions at 2 L bioreactor scale.** Glucose starvation conditions were realized using a DO signal-based pulse feeding strategy adding 0.4 g glucose per liter on demand upon carbon source depletion. Feed start after 24h. Spikes in dissolved Oxygen were used to trigger feed pulses. Dissolved Oxygen (pO_2_) is shown in blue and Oxygen Uptake Rate (OUR) is shown in red.

**
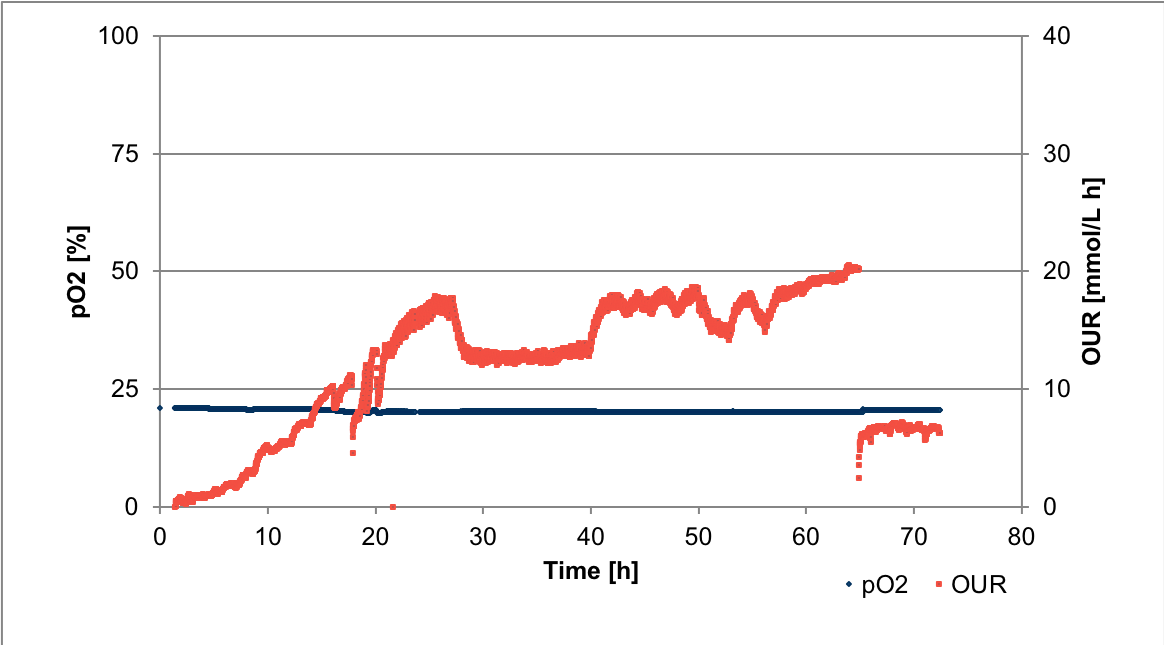
**

**Figure S5 Time profiles of dissolved Oxygen and Oxygen Uptake during excess glucose conditions at 2 L bioreactor scale** Excess glucose was achieved via a semi-continuous feeding strategy with fixed glucose delivery of 4 g glucose per liter per hour. Dissolved Oxygen (pO_2_) is shown in blue and Oxygen Uptake Rate (OUR) is shown in red.

| **A** | ATGACCCTGCAGGAAACCAGCGTTCTGGAACCAACCTTGCAGGGCACCACCACCCTGCCAGGTCTGCTGGCTCAACGTGTTGCAGAACATCCGGAAGCTATTGCAGTGGCGTACCGTGATGACAAACTGACCTTTCGTGAACTGGCGAGCCGTAGCGCGGCGCTGGCGGACTATCTGGAACACCTGGGTGTTAGCGCGGATGACTGCGTGGGTCTGTTCGTTGAACCGAGCATTGATCTGATGGTGGGTGCATGGGGTATCCTGAATGCTGGTGCAGCTTACCTGCCGCTGAGCCCAGAATATCCGGAAGATCGTCTGCGTTACATGATTGAAAACAGCGAAACCAAAATTATCCTGGCTCAACAGCGTCTGGTTAGCCGTCTGCGTGAACTGGCACCGAAGGACGTGACCATCGTTACCCTGCGTGAAAGCGAAGCGTTTGTTCGTCCGGAAGGCACCGAAGCTCCGGCTGCACGTAGCGCACGTCCGGATACCCTGGCGTACGTGATCTATACCAGCGGTAGCACCGGCAAACCGAAGGGTGTTATGATTGAACATCGTAGCATCGTGAATCAACTGGGTTGGCTGCGTGAAACCTACGCTATTGACCGTAGCAAAGTTATCCTGCAGAAGACCCCGATGAGCTTCGATGCGGCTCAATGGGAAATTCTGAGCCCGGCTAATGGTGCAACCGTTGTGATGGGTGCTCCGGGTGTGTATGCAGACCCGGAAGGTCTGATTGAAACCATCGTGAAACACAACGTTACCACCCTGCAATGCGTTCCGACCCTGCTGCAGGGTCTGATCGATACCGAAAAGTTTCCGGAATGTGTGAGCCTGCAACAGATTTTCAGCGGTGGTGAAGCTCTGAGCCGTCTGCTGGCAATCCAAACCACCCAGGAAATGCCGGGTCGTGCACTGATTAATGTTTACGGTCCGACCGAAACCACCATTAATAGCAGCAGCTTTCCGGTGGACCCGGCTGATCTGGACGAAGGTCCGCAGAGCATTAGCATCGGTAGCCCGGTTCATGGCACCACCTATCACATTCTGGATAAAGAAACCCTGAAGCCGGTGGGTGTTGGTGAAATCGGTGAACTGTATATTGGTGGTATCCAACTGGCACGTGGTTACCTGCATCGTGATGACCTGACCGCTGAACGTTTTCTGGAAATTGAACTGGAAGAAGGTGCTGAGCCGGTTCGTCTGTACAAAACCGGCGATCTGGGTCAATGGAATAACGATGGCACCGTGCAGTTCGCGGGTCGTGCTGATAACCAAGTTAAGCTGCGTGGTTATCGTGTGGAACTGGACGAAATTAGCCTGGCGATCGAAAATCATGATTGGGTTCGTAACGCGGCGGTTATTGTTAAAAATGACGGTCGTACCGGCTTTCAGAACCTGATTGCTTGTATCGAACTGAGCGAAAAGGAAGCTGCACTGATGGATCAAGGTAATCACGGTAGCCATCATGCGAGCAAGAAAAGCAAACTGCAAGTTAAGGCACAGCTGAGCAACCCGGGTCTGCGTGATGACGCTGAACTGGCGGCTCGTCCGGCGTTCGATCTGGAAGGTGCTGAACCGACCCCGGAACAGCGTGCGCGTGTGTTTGCTCGTAAAACCTACCGTTTCTATGAAGGTGGTGCAGTTACCCAAGCGGACCTGCTGGGTCTGCTGGGTGCTACCGTGACCGCAGGTTACAGCCGTAAGGCAGCGGATCTGGCTCCGGCAGAACTGGGTCAAATTCTGCGTTGGTTTGGCCAGTATATCAGCGAAGAACGTCTGCTGCCGAAATACGGTTATGCGAGCCCGGGTGCACTGTACGCGACCCAAATGTATTTCGAACTGGAAGGTGTTGGTGGTCTGAAGCCGGGTTACTATTACTATCAGCCGGTTCGTCATCAGCTGGTTCTGATTAGCGAACGTGAAGCTACCGGCAAAGCGACCGCTCAGATTCACTTTATCGGTAAAAAGAGCGGTATCGAGCCGGTTTACAAGAATAACATTCTGGAAGTGCTGGAAATCGAAACCGGCCACATGGTTGGTCTGTTCGAACAAATTCTGCCGGCGTATGGTCTGGACATCCACGATCGTGCGTACGAACCGGCTGTTAAAGACCTGCTGGATGTGGCTGATGAAGACTACTATCTGGGCACCTTTGAACTGGTTCCGCATGCAGGTGCGCGTGATGACCAAGCGGAAGTGTACGTTCAGACCCACGGTGGTAAAGTGGCTGGTCTGCCGGAAGGTCAATACCGTTATGAAAATGGTGAACTGACCCGTTTTAGCGATGACATTGTGCTGAAAAAGCATGTTATTGCTATCAACCAAAGCGTTTATCAGGCTGCGAGCTTTGGTATTAGCGTGTACAGCCGTGCAGAAGAAGAATGGCTGAAATATATCACCCTGGGTAAAAAGCTGCAGCACCTGATGATGAATGGTCTGAACCTGGGTTTCATGAGCAGCGGTTACAGCAGCAAAACCGGCAATCCGCTGCCAGCAAGCCGTCGTATGGACGCAGTTCTGGGTGCGAACGGTGTGGATAGCGCGCCGATGTACTTTTTCGTTGGTGGTCGTATTAGCGACGAACAAATCGGTCATGAGGGTATGCGTGAAGATAGCGTTCACATGCGTGGTCCGGCGGAACTGATTCGTGATGACCTGGTGAGCTTTCTGCCGGACTACATGATCCCGAATCGTGTTGTGGTTTTCGATCGTCTGCCGCTGAGCGCGAACGGTAAAATTGACGTTAAGGCTCTGGCGGCGAGCGATCAAGTGAATGCGGAACTGGTTGAACGTCCGTTCGTGGCTCCGCGTACCGAAACCGAAAAAGAAATCGCAGCGGTTTGGGAAAAGGCACTGCGTCGTGAAAACGCGAGCGTGCAAGATGACTTTTTCGAAAGCGGTGGTAATAGCCTGATTGCAGTGGGTCTGGTTCGTGAACTGAACGCGCGTCTGGGTGTTAGCCTGCCGCTGCAGAGCGTGCTGGAAAGCCCGACCATCGAAAAACTGGCACGTCGTCTGGAACGTGAAGTTGCGCAAGAAAGCAGCCGTTTTGTGCGTCTGCATGCGGAAACCGGCAAAGCTCGTCCGGTTATTTGCTGGCCGGGTCTGGGTGGTTACCCGATGAATCTGCGTAGCCTGGCGGGTGAAATCGGTCTGGGTCGTAGCTTCTACGGTGTGCAGAGCTATGGTATTAACGAAGGTGAAACCCCGTATGAAACCATCACCGAAATGGCTAAAAAGGACATTGAAGCACTGAAAGAAATCCAACCGGCTGGTCCGTACACCCTGTGGGGTTATAGCTTTGGTGCTCGTGTTGCATTCGAAACCGCGTACCAACTGGAACAGGCTGGTGAAAAGGTGGATAACCTGTTTCTGATTGCTCCGGGTAGCCCGAAAGTTCGTGCAGAAAATGGTAAAGTGTGGGGTCGTGAAGCGAGCTTCGCTAACCGTGGTTATACCACCATTCTGTTTAGCGTTTTTACCGGCACCATTAGCGGTCCGGATCTGGACCGTTGTCTGGAAACCGTGACCGACGAAGCGAGCTTTGCGGAGTTTATTAGCGAACTGAAAGGTATCGATGTTGACCTGGCGCGTCGTATTATCAGCGTGGTTGGTCAGACCTACGAATTTGAATATAGCTTCCACGAACTGGCAGAACGTACCCTGCAAGCGCCGATTAGCATCTTTAAGGCTGTTGGTGACGACTATAGCTTCCTGGAAAACAGCAGCGGTTACAGCGCGGAACCGCCGACCGTTATCGATCTGGACGCTGATCATTACAGCCTGCTGCGTGAAGATATTGGTGAACTGGTGAAACACATCCGTTATCTGCTGGGTGAA |
| --- | --- |
| **B** | **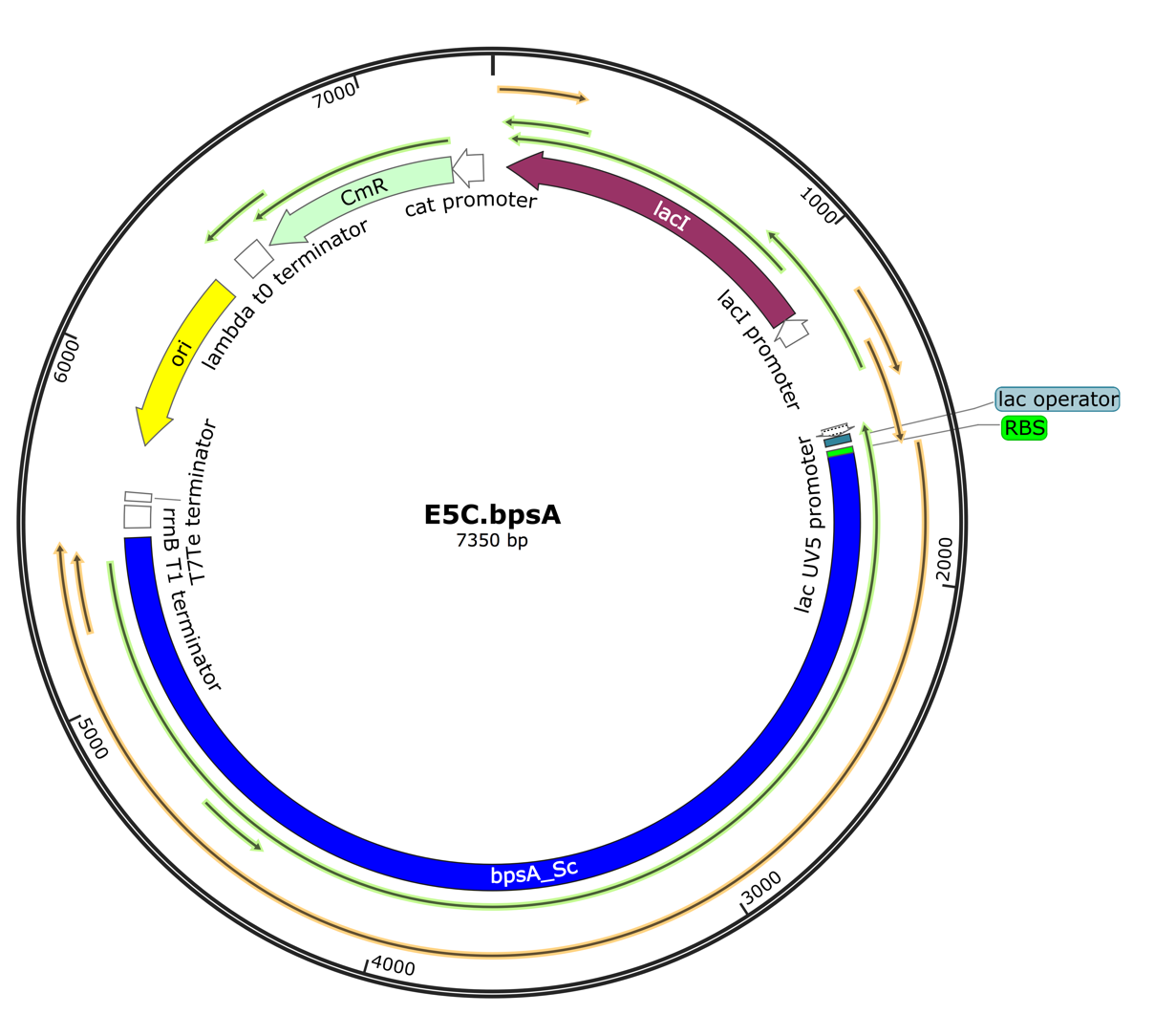** |

**Figure S6 DNA sequences and constructs used in this study**

**A** Sequence of *Streptomyces lavendulae* *bpsA* codon optimized for expression in *S. cerevisiae***. B** The codon-optimized version was cloned into an E5C plasmid backbone under the control of an IPTG inducible lacUV5 promoter for production of Indigoidine in *E. coli.*

.


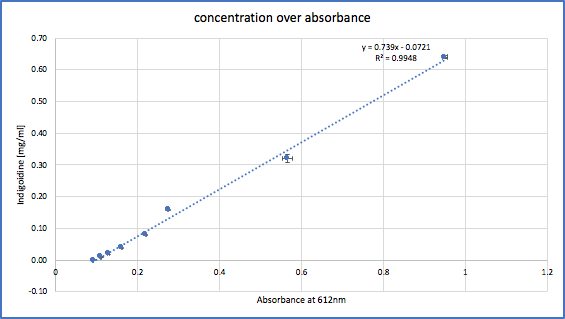


**Figure S7 Standard curve of Indigoidine absorbance at 612 nm in DMSO.**

Absorbance values were obtained for serial dilutions of purified Indigoidine in DMSO. The equation for the trendline is y= 0.739x – 0.0721, R^2^= 0.9948
