# Supplementary material for: Correction to: Production efficiency of the bacterial non-ribosomal peptide indigoidine relies on the respiratory metabolic state in S. cerevisiae
Source: Microb Cell Fact. 2019 Dec 29;18:218. doi: 10.1186/s12934-019-1262-2 (PMC6936116; doi:10.1186/s12934-019-1262-2)
Supplement: Supplementary file 1 — Additional file 1: Figure S7. Standard curve of Indigoidine absorbance at 612 nm in DMSO. Absorbance values were obtained for serial dilutions of purified Indigoidine in DMSO. The equation for the trendline is: y = 0.152x − 0.111 R2 = 0.9986. Indigoidine was purified from microbial cultures per Yu et al. (https://doi.org/10.1007/s10295-012-1207-9). Figure shows one representative plot (of three), each with measurements in triplicate. [file 12934_2019_1262_MOESM1_ESM.docx]

**Figure S7:**

**Figure S7 Standard curve of Indigoidine absorbance at 612 nm in DMSO.**

Absorbance values were obtained for serial dilutions of purified Indigoidine in DMSO. The equation for the trendline is: y= 0.152x- 0.111 R^2^=0.9986. Indigoidine was purified from microbial cultures per Yu et al (doi:10.1007/s10295-012-1207-9.). Figure shows one representative plot (of three), each with measurements in triplicate.
